# Lactylation profiling reveals novel biomarkers and immune interactions in pancreatic cancer

**DOI:** 10.1016/j.tranon.2026.102710

**Published:** 2026-02-23

**Authors:** Dong Xu, Qiuyang Chen, Liang Wang, Liangliang Li, Kangwen Cheng, Xinchun Liu, Kai Lin

**Affiliations:** a Department of Pancreatic and Metabolic Surgery, Nanjing Drum Tower Hospital, Affiliated Hospital of Medical School, Nanjing University, Nanjing 210008, Jiangsu Province, China; b Collaborative Innovation Center for Cancer Personalized Medicine and Hepatobiliary Center, The First Hospital Affiliated with Nanjing Medical University, Nanjing 210029, Jiangsu Province, China; c Department of Gastrointestinal and Anal Surgery, Affiliated Hangzhou First People's Hospital, School of Medicine, Westlake University, Hangzhou 310000, Zhejiang Province, China; d The Fourth Clinical Medical College, Zhejiang Chinese Medical University, Hangzhou 310053, Zhejiang Province, China; e The First Clinical Medical College, Zhejiang Chinese Medical University, Hangzhou 310053, Zhejiang Province, China; f Department of Anorectal Surgery, The First Affiliated Hospital of Zhejiang Chinese Medical University (Zhejiang Provincial Hospital of Chinese Medicine), Hangzhou 310006, Zhejiang Province, China

**Keywords:** Pancreatic cancer, Protein lactylation, Biomarkers, Immune regulation, Pathway analysis

## Abstract

Pancreatic cancer, known for its aggressive nature and poor prognosis, often eludes early detection and effective treatment. This study investigates the underexplored terrain of protein lactylation in pancreatic cancer to identify potential biomarkers and understand its immunological implications. Proteomic and transcriptomic analyses were conducted to profile lactylation changes, identifying 11 upregulated and 1 downregulated lactylation-related proteins. Intersection analysis between proteomics and transcriptomics highlighted four key genes (COQ9, GAA, LYST and TP53) with consistent expression trends, suggesting their central role in the lactylation pathway within pancreatic cancer. ROC curve analysis underscored the diagnostic potential of GAA and LYST, with AUCs over 0.9. Further, ssGSEA revealed significant correlations between these core genes and various immune cells, indicating an immune-modulatory role. Notably, most core lactylation-related genes were positively associated with immune checkpoint molecules, barring COQ9. GSEA of gene expression groups delineated three conserved upregulated KEGG pathways, with additional REACTOME pathway analysis uncovering 68 conserved pathways. These findings highlight lactylation’s involvement in pancreatic cancer progression and its possible exploitation for diagnostic and therapeutic advancements.

## Highlight

• First characterize the global lactylation profiling in pancreatic cancer tissues, identifying metabolism- and immune-related differential lactylation sites.
• Discover and validate novel lactylation-derived biomarkers for predicting prognosis and immunotherapy response in pancreatic cancer patients independently.
• Uncover a new mechanism of lactylation regulating the tumor immune microenvironment in pancreatic cancer, providing new targets for TIME-targeted therapies.

## Graphical abstract

**Figure:**
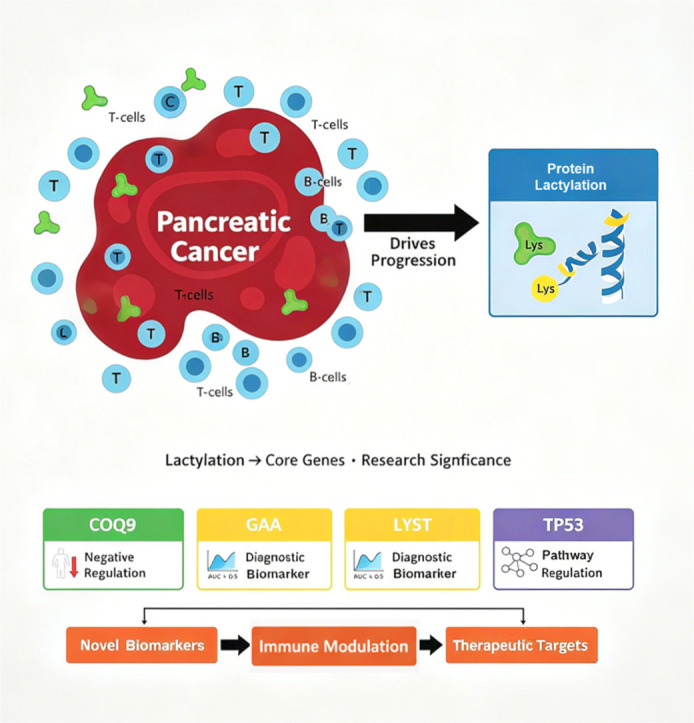

## Introduction

The intricate interplay between metabolic reprogramming and cancer progression has long been a subject of intense research within the oncological community. Among the various metabolic alterations observed in cancer, the phenomenon of lactylation, a post-translational modification where lactate adds to lysine residues on proteins, has recently emerged as a significant area of interest [1]. This modification, previously underexplored, has been found to play critical roles in various cellular processes, including gene expression regulation, immune response modulation, and cancer metabolism [2]. Given the pervasive nature of metabolic reprogramming in cancer cells, characterized by heightened glucose uptake and lactate production even in the presence of oxygen (the Warburg effect), understanding the implications of lactylation in the oncogenic context is of paramount importance [2]. Clinical ¹⁸F-FDG PET studies show that glucose uptake in PDAC is typically 2- to 3-fold higher than in normal pancreatic tissue. Moreover, metabolomic and functional studies demonstrate that lactate production is increased by 4- to 5-fold in PDAC cells and tumor tissues compared to normal counterparts, driven by oncogenic KRAS signaling and hypoxia-induced metabolic reprogramming [[3], [4], [5], [6]].

Pancreatic cancer, particularly pancreatic ductal adenocarcinoma (PDAC), stands as one of the most lethal malignancies worldwide, with dismal survival rates that have seen little improvement over the past decades. This grim prognosis is largely due to the disease's asymptomatic nature in its early stages, leading to late diagnoses, and the profound resistance of pancreatic tumors to conventional therapies [7,8]. The tumor microenvironment of pancreatic cancer, characterized by dense stromal reactions, hypoxia, and nutrient deprivation, further complicates therapeutic interventions, underscoring the urgent need for novel therapeutic targets and strategies [9,10].

The concept of lactylation in the context of pancreatic cancer offers a novel lens through which the disease's metabolic and immunological landscape can be re-examined [11]. Recent studies have highlighted lactylation's potential role in modulating immune responses, influencing gene expression related to cancer cell proliferation [12], metastasis, and adaptation to the tumor microenvironment. These revelations point towards lactylation not merely as a byproduct of altered cancer metabolism but as a pivotal regulator of cancer biology [13].

Given the nascent state of research in this field, our study aims to bridge the gap in understanding the role of lactylation in pancreatic cancer comprehensively. By integrating proteomic and transcriptomic analyses [14], we seek to unravel the complex network of lactylation-modified proteins and their gene counterparts in pancreatic cancer, elucidating their potential roles in tumor progression, metastasis, and response to therapy [15]. This approach not only allows for a deeper understanding of the biological processes influenced by lactylation in pancreatic cancer but also aids in identifying novel biomarkers and therapeutic targets [16].

Pancreatic cancer's notoriously immunosuppressive microenvironment poses a significant challenge to immunotherapy, a hurdle that may be addressed by exploring the interplay between protein lactylation and the immune system [16]. Within this immunosuppressive microenvironment, the proportion of regulatory T cells (Tregs) is 3-4 times higher than in normal tissues, and M2 macrophages constitute over 60 % of total macrophages, significantly inhibiting effector T cell function [[17], [18], [19]]. Our study delves into this connection, analyzing how lactylation-modified proteins correlate with immune cell presence and activity within the tumor milieu, revealing potential targets for novel immunotherapeutic strategies [20]. This research advances our comprehension of pancreatic cancer's complex biology and progression, spotlighting lactylation's influence on metabolic reprogramming, immune evasion, and gene regulation [21]. Ultimately, these insights offer a beacon of hope for identifying innovative biomarkers and treatments, aiming to enhance the prognosis for this formidable disease.

## Methods

### Proteomic and transcriptomic data analysis

CFPAC-1, MIAPaCa-2, and hTERT-HPNE cells were cultured in DMEM supplemented with 10 % fetal bovine serum (FBS, Gibco) and 1 % penicillin-streptomycin (Solarbio). Cells were maintained in a humidified incubator at 37°C with 5 % CO2, and cells in the logarithmic growth phase were selected for protein extraction and subsequent experiments.L-lactate (Sigma-Aldrich, L7907, purity ≥98 %) and 2-deoxy-D-glucose (2-DG, Sigma-Aldrich, D8375, purity ≥99 %) were used.

Proteomic analysis in this study was based on in-house protein data, while transcriptomic data incorporated two primary sources: the TCGA-PAAD dataset with 177 pancreatic cancer samples and the GTEx database with 167 normal pancreatic samples. Raw transcriptomic data (FASTQ format) were assessed for quality using FastQC (version 0.11.9). Subsequently, Trimmomatic (version 0.39) was employed for quality control: reads containing adapter sequences were removed, reads with a Q30 < 80 % (base quality ≥ 30 accounting for less than 80 %) were filtered out, and reads shorter than 50 bp were discarded. The clean data were aligned to the human reference genome (GRCh38.p13) using HISAT2, ensuring an alignment rate of ≥90 % for downstream differential analysis. Lactylation-related genes were identified by searching GSEA databases (MSigDB v7.5.1: Canonical Pathways, GO Biological Processes, Reactome) using keywords such as 'lactylation', 'lactate metabolism', and 'lysine lactylation'. Irrelevant pathways (e.g., lactose metabolism) were excluded. The selection was validated against core lactylation pathways reported in recent literature, resulting in a unique set of 329 genes from 10 confirmed pathways. Visualization of data included volcano plots created using the ggVolcano and ggrepel packages, and Venn diagrams generated through Finrich software (version 3.1.3). Enrichment analyses were conducted using the clusterProfiler package, with subsequent clustering and visualization of GO enrichment facilitated by the aPEAR package and the cols4all package. KEGG pathway insights were depicted via bubble and chord diagrams using ggplot2 and GOplot packages, respectively.

Additionally, protein-protein interaction networks were analyzed using the STRING database and visualized with Cytoscape software (version 3.9.1), employing the MCODE algorithm to identify significant interactive modules. A correlation coefficient threshold > 0.7 was selected as it corresponds to the 'high confidence' level in the STRING database and aligns with standard thresholds for oncology PPI network analysis, effectively excluding low-confidence non-specific interactions.

### Clinical and immune analysis integration

Clinical data pertaining to pancreatic cancer was sourced from the TCGA database, with visualization managed through the ggpubr package. Inclusion criteria for TCGA-PAAD clinical data were: pathological diagnosis of PDAC, complete clinical staging information (T/N/M), follow-up duration ≥ 6 months, and exclusion of patients with other concurrent malignancies. A total of 177 eligible samples were included. Immune cell abundance and activity assessments were performed using GSVA and GSEABase packages, while correlations with lactylation-related genes were visually represented in radar charts by employing the fmsb package [22,23]. ssGSEA was performed using the GSVA package (version 1.44.2) with gene set size thresholds set to a minimum of 10 and a maximum of 500. Differential gene expression analysis was executed using the limma package (version 3.52.2) with screening thresholds of adj.P < 0.05 and |log2FC| > 1. Heatmaps to depict correlation matrices were constructed using the ggplot2 package. Gene Set Enrichment Analysis (GSEA) was executed through the limma package for differential gene expression analysis and the clusterProfiler for enrichment analysis, with results visualized using the GseaVis package. For integrative data representation, Venn diagrams were produced using an accessible online tool, providing a straightforward comparative view of the datasets.

## Results

### Differential analysis in proteomics

Proteomic differential analysis aims to identify variations in protein expression levels between different conditions or groups. In this case, the focus is on comparing CFPAC-1/ hTERT-HPNE and MIAPaCa-2/ hTERT-HPNE groups. Protein expression ratios are expressed in log2 scale, where log2(CFPAC-1/ hTERT-HPNE +1) or log2(MIAPaCa-2/ hTERT-HPNE +1) values greater than 2 indicate upregulation, and values less than 0.5 signify downregulation. To ensure consistent sample size throughout the analysis, preliminary quality control was conducted to retain only those proteins detected in both comparison groups, resulting in a total of 7664 proteins (see Supplementary Table 1). Quality control criteria included retaining proteins with ≥ 2 peptide matches, ≥ 10 % protein coverage, and a detection rate of ≥ 80 % in both replicate groups.

### Visualization of differential protein expression

**Fig. 1:**
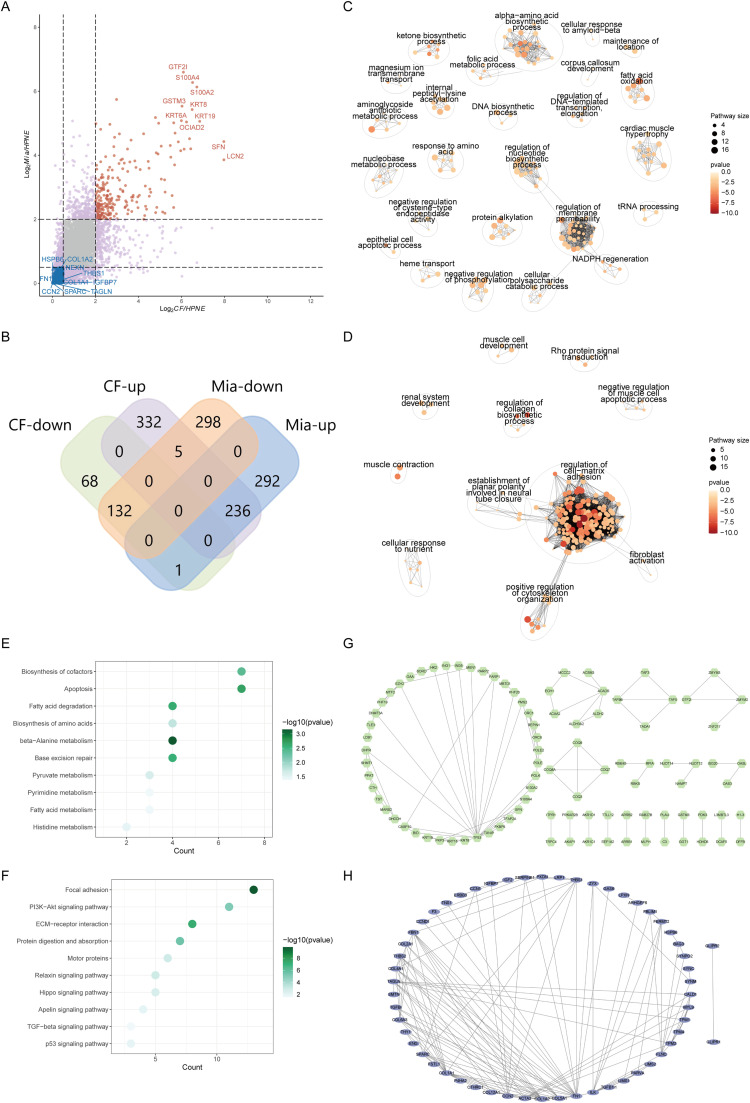
Differential proteomic analysis of CFPAC-1/ hTERT-HPNE and MIAPaCa-2/ hTERT-HPNE comparisons. A. Scatter plot displaying the regulation of proteins across CFPAC-1/ hTERT-HPNE and MIAPaCa-2/ hTERT-HPNE groups. Red dots represent genes upregulated in both CFPAC-1/ hTERT-HPNE and MIAPaCa-2/ hTERT-HPNE, blue dots indicate genes downregulated in both groups, purple dots show genes either upregulated or downregulated in one of the two groups, and grey dots denote genes without significant regulation in either group. B. Venn diagram quantifying the intersection of regulated genes between CFPAC-1/ hTERT-HPNE and MIAPaCa-2/ hTERT-HPNE groups. It highlights genes that are commonly upregulated (236 genes) and downregulated (132 genes) in both groups, as well as genes uniquely upregulated or downregulated in CFPAC-1/ hTERT-HPNE (332 and 68 genes, respectively) and MIAPaCa-2/ hTERT-HPNE (292 and 236 genes, respectively). C. GO enrichment and clustering analysis of genes upregulated in both CFPAC-1/ hTERT-HPNE and MIAPaCa-2/ hTERT-HPNE groups, revealing core biological process modules associated with membrane permeability, nucleotide biosynthetic process. D. GO enrichment and clustering analysis of genes downregulated in both CFPAC-1/ hTERT-HPNE and MIAPaCa-2/ hTERT-HPNE groups, showing core biological process modules linked to the regulation of cell matrix adhesion and cytoskeletal assembly. E. KEGG enrichment analysis of genes commonly upregulated in both CFPAC-1/ hTERT-HPNE and MIAPaCa-2/ hTERT-HPNE groups, with involvement in the biosynthesis of cofactors, apoptosis and fatty acid degradation signaling pathways. F. KEGG enrichment analysis of genes commonly downregulated in both CFPAC-1/ hTERT-HPNE and MIAPaCa-2/ hTERT-HPNE groups, associated with the focal adhesion, PI3K-Akt pathways and ECM receptor interaction signaling pathways. G-H. Protein-protein interaction analyses performed by the STRING algorithm on genes commonly upregulated (G) and downregulated (H) in both groups, with a correlation coefficient threshold greater than 0.7 to identify strongly related core protein interaction modules.

Scatter plots are used to visualize differential protein expression, with red indicating genes upregulated in both CFPAC-1/ hTERT-HPNE and MIAPaCa-2/ hTERT-HPNE, blue representing genes downregulated in both groups, purple for genes upregulated or downregulated in either CFPAC-1/ hTERT-HPNE and MIAPaCa-2/ hTERT-HPNE, and grey for genes that are neither upregulated nor downregulated in both comparison groups (Fig. 1A). Venn diagrams provide a statistical overview of the gene intersections, identifying 236 genes commonly upregulated and 132 genes commonly downregulated in both HPEN/CF and Mia/CF. Additionally, the diagrams show genes uniquely upregulated or downregulated within each group (Fig. 1B).

### Common characteristics in CFPAC-1/hTERT-HPNE and MIAPaCa-2/ hTERT-HPNE comparisons

An analysis of the common characteristics between CFPAC-1/ hTERT-HPNE and MIAPaCa-2/ hTERT-HPNE comparisons involved conducting GO enrichment analysis and clustering on genes upregulated in both groups. This revealed core modules associated with membrane permeability, nucleotide biosynthetic process (Fig. 1C). In contrast, genes commonly downregulated were primarily related to the regulation of cell matrix adhesion and cytoskeletal assembly (Fig. 1D). KEGG enrichment analysis showed that commonly upregulated genes were involved in the biosynthesis of cofactors, apoptosis and fatty acid degradation signaling pathways (Fig. 1E), whereas commonly downregulated genes were mainly involved in the focal adhesion, PI3K-Akt pathways and ECM receptor interaction signaling pathways (Fig. 1F). Protein-protein interaction analyses using the STRING algorithm, with a correlation coefficient threshold set above 0.7 (strong correlation), identified core protein interaction modules potentially regulating CFPAC-1 and MIAPaCa-2 through common herbal proteins (Fig. 1G-H).

### CFPAC-1/ hTERT-HPNE group-specific analysis

**Fig. 2:**
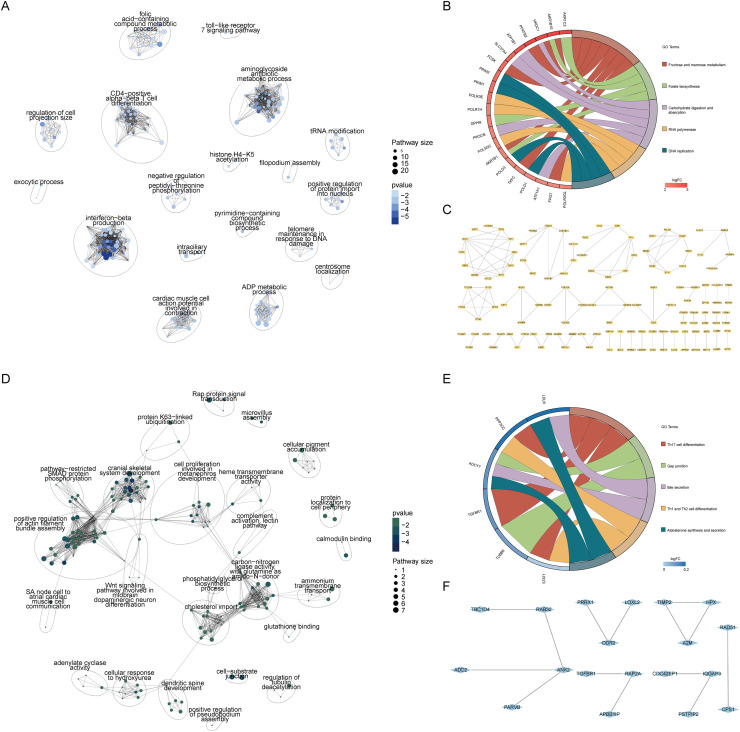
Group-specific analysis for CFPAC-1/ hTERT-HPNE comparison. A. GO enrichment and clustering analysis for genes uniquely upregulated in the CFPAC-1/ hTERT-HPNE comparison group, revealing central biological processes such as the CD4 positive alpha-beta T cell differentiation, interferon-beta production and aminoglycoside antibiotic metabolic process. B. KEGG enrichment analysis showing the top five pathways for CFPAC-1/ hTERT-HPNE upregulated genes, including the fructose and mannose metabolism, folate biosynthesis, carbohydrate digestion and absorption, RNA polymerase and DNA replication pathway. C. Protein-protein interaction analysis with a correlation coefficient threshold greater than 0.7, highlighting the initial interaction network and, after further processing with the MCODE algorithm, revealing core sub-network modules that represent potential regulatory network proteins independently associated with CFPAC-1 upregulated genes. D. GO enrichment and clustering analysis for genes uniquely downregulated in the CFPAC-1/ hTERT-HPNE comparison group, identifying key biological processes such as positive regulation of actin filament bundle assembly, carbon-nitrogen ligase activity with glutamine as an amido-N-donor, cranial skeletal system development and cholesterol import. E. KEGG enrichment analysis displaying the top five pathways for CFPAC-1/ hTERT-HPNE downregulated genes, including Th17 cell differentiation, gap junction, bile secretion, Th1 and Th2 cell differentiation, aldosterone synthesis and secretion pathway. F. Protein-protein interaction analysis using the same correlation coefficient threshold as in C, which delineates the initial network and, following further analysis with MCODE, exposes three core sub-network modules that represent potential regulatory network proteins independently associated with CFPAC-1 downregulated genes.

The GO enrichment analysis of the CFPAC-1/ hTERT-HPNE comparison group specifically focused on upregulated genes, identifying core biological process modules related to CD4 positive alpha-beta T cell differentiation, interferon-beta production and aminoglycoside antibiotic metabolic process (Fig. 2A). The top five pathways from the KEGG enrichment analysis included the fructose and mannose metabolism, folate biosynthesis, carbohydrate digestion and absorption, RNA polymerase and DNA replication pathway (Fig. 2B). Protein interaction analysis, using a correlation coefficient threshold greater than 0.7, provided an initial interaction network. Further analysis with the MCODE algorithm extracted core sub-network modules, potentially representing regulatory network proteins independently involved with CFPAC-1 upregulated genes (Fig. 2C).

A similar GO enrichment analysis on the CFPAC-1/ hTERT-HPNE group's downregulated genes revealed core biological process modules related to positive regulation of actin filament bundle assembly, carbon-nitrogen ligase activity with glutamine as an amido-N-donor, cranial skeletal system development and cholesterol import (Fig. 2D). The top five pathways from KEGG enrichment analysis included Th17 cell differentiation, gap junction, bile secretion, Th1 and Th2 cell differentiation, aldosterone synthesis and secretion pathway (Fig. 2E). Protein interaction analysis, with the same correlation threshold, yielded an initial network that, when further analyzed with MCODE, revealed core sub-network modules, potentially indicative of regulatory network proteins independently involved with CFPAC-1 downregulated genes (Fig. 2F).

### MIAPaCa-2/ hTERT-HPNE group-specific analysis

**Fig. 3:**
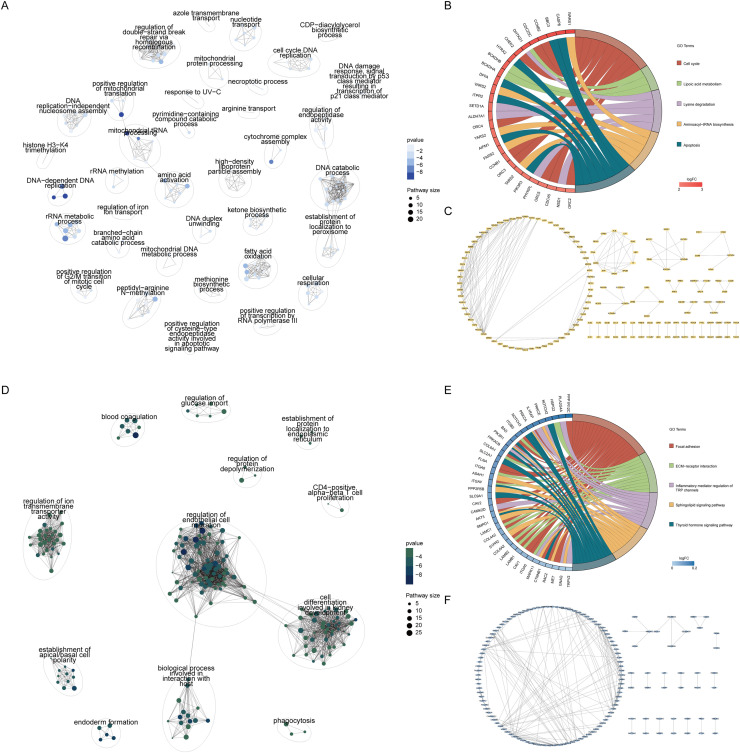
Group-specific analysis for MIAPaCa-2/ hTERT-HPNE comparison. A. GO enrichment and clustering analysis for genes uniquely upregulated in the Mia/CF comparison group, identifying central biological processes such as the double-strand break repair via homologous recombination, DNA independent DNA replication and DNA catabolic process. B. KEGG enrichment analysis indicating the top five pathways for Mia/CF upregulated genes, including cell cycle, lipoic acid metabolism, lysine degradation, aminoacyl-tRNA biosynthesis, and apoptosis pathway. C. Protein-protein interaction analysis with a correlation coefficient threshold greater than 0.7, presenting the initial interaction network and, after further refinement with the MCODE algorithm, uncovering three core sub-network modules indicative of potential regulatory network proteins independently associated with MIAPaCa-2 upregulated genes. D. GO enrichment and clustering analysis for genes uniquely downregulated in the Mia/CF comparison group, highlighting essential biological processes like regulation of endothelial cell migration and ion transmembrane transporter activity. E. KEGG enrichment analysis showcasing the top five pathways for Mia/CF downregulated genes, which include focal adhesions, ECM receptor interaction, inflammatory mediator regulation of TRP channels, sphingolipid signaling pathway and thyroid hormone signaling pathway. F. Protein-protein interaction analysis using the same correlation coefficient threshold as in C, which yields the initial network and, following further analysis with MCODE, reveals three core sub-network modules likely representing regulatory network proteins independently associated with MIAPaCa-2 downregulated genes.

The GO enrichment analysis for the MIAPaCa-2/ hTERT-HPNE comparison group, focusing on uniquely upregulated genes, identified core biological process modules related to the double-strand break repair via homologous recombination, DNA independent DNA replication and DNA catabolic process (Fig. 3A). The top five pathways from KEGG enrichment analysis were cell cycle, lipoic acid metabolism, lysine degradation, aminoacyl-tRNA biosynthesis, and apoptosis pathway (Fig. 3B). Protein interaction analysis, using a correlation coefficient threshold greater than 0.7, led to an initial interaction network. Further analysis with MCODE identified core sub-network modules, suggesting regulatory network proteins independently involved with MIAPaCa-2 upregulated genes (Fig. 3C).

Similarly, GO enrichment analysis of the MIAPaCa-2/ hTERT-HPNE group's downregulated genes revealed core biological process modules related to regulation of endothelial cell migration and ion transmembrane transporter activity (Fig. 3D). The top five pathways from KEGG enrichment analysis included focal adhesions, ECM receptor interaction, inflammatory mediator regulation of TRP channels, sphingolipid signaling pathway and thyroid hormone signaling pathway (Fig. 3E). Protein interaction analysis, with the same correlation threshold, yielded an initial network that, when further analyzed with MCODE, revealed core sub-network modules, potentially representing regulatory network proteins independently involved with MIAPaCa-2 downregulated genes (Fig. 3F).

### Lactylation and cancer

**Fig. 4:**
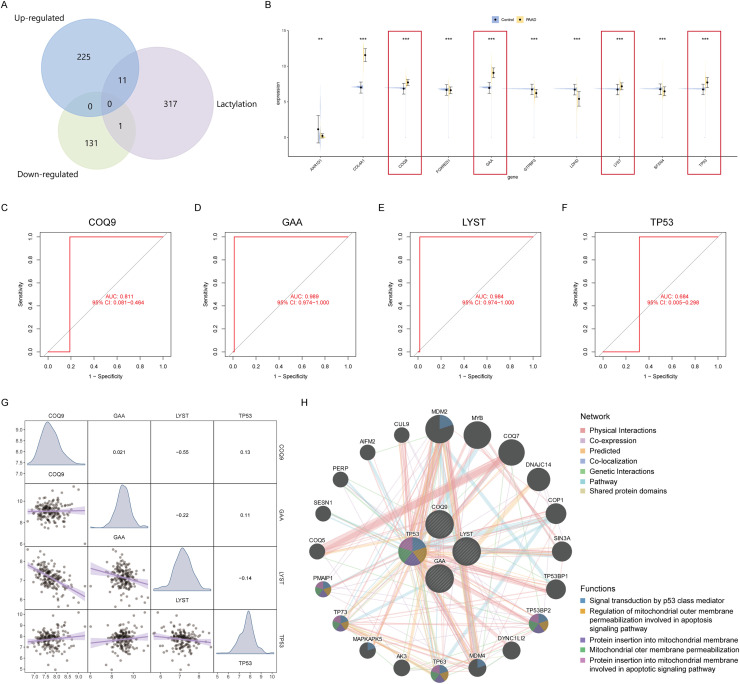
Intersection analysis and diagnostic potential of lactylation-related proteins and genes in pancreatic cancer. A. Venn diagram illustrating the intersection of lactylation-related proteins that are commonly upregulated (11 proteins) and downregulated (1 protein) in CFPAC-1/ hTERT-HPNE and MIAPaCa-2/ hTERT-HPNE comparison groups. B. Semi-violin plots representing the gene-level expression of 10 differentially lactylated proteins in pancreatic cancer compared to control samples. Four genes (COQ9, GAA, LYST and TP53) show expression trends consistent with their protein levels, highlighting their potential significance in pancreatic cancer lactylation pathways. C-F. Receiver operating characteristic (ROC) curves demonstrating the diagnostic power of lactylation-related genes, with GAA and LYST showing areas under the curve (AUC) greater than 0.9, suggesting their strong potential as biomarkers for pancreatic cancer. G. Correlation matrix depicting the expression relationships between the five core lactylation-related genes, with significant negative correlations between COQ9 and GAA, and LYST and COQ9, indicating their potential interaction in the disease process. H. GENEMANIA network analysis showing the protein interactions and involved signaling pathways of the four core lactylation-related genes, providing insights into their functional associations and potential roles in pancreatic cancer progression.

The relationship between lactylation and cancer has been extensively reported in the literature. An intersection analysis was performed on the proteins that are commonly upregulated and downregulated, with a focus on those related to lactylation. This analysis identified 11 upregulated lactylation-related proteins and 1 downregulated lactylation-related protein (Fig. 4A). To further pinpoint the core lactylation-related genes in pancreatic cancer, an analysis incorporating transcriptomics was conducted in addition to proteomics. Due to the limited number of normal tissue samples in TCGA-PAAD, 167 normal pancreatic samples were obtained from the GTEx database, and batch correction was performed using the 'combat' function of the 'svm' package. Violin plots illustrated the gene-level expression of 10 differential lactylation proteins, with only 4 genes (COQ9, GAA, LYST and TP53) showing expression trends consistent with the protein expression trends, suggesting their importance in pancreatic cancer lactylation-related protein expression (Fig. 4B).

### Diagnostic potential of lactylation-related genes

ROC curve analysis demonstrated that GAA and LYST have good diagnostic efficacy for pancreatic cancer, with areas under the curve (AUC) both exceeding 0.9 (Fig.s 4C-F). Expression correlation analysis revealed significant negative correlations between COQ9 and GAA, and LYST and COQ9 (Fig. 4G). GENEMANIA provided insights into the protein interactions and involved signaling pathways of the 4 core lactylation-related genes (Fig. 4H).

### Clinical relevance of lactylation-related genes

**Fig. 5:**
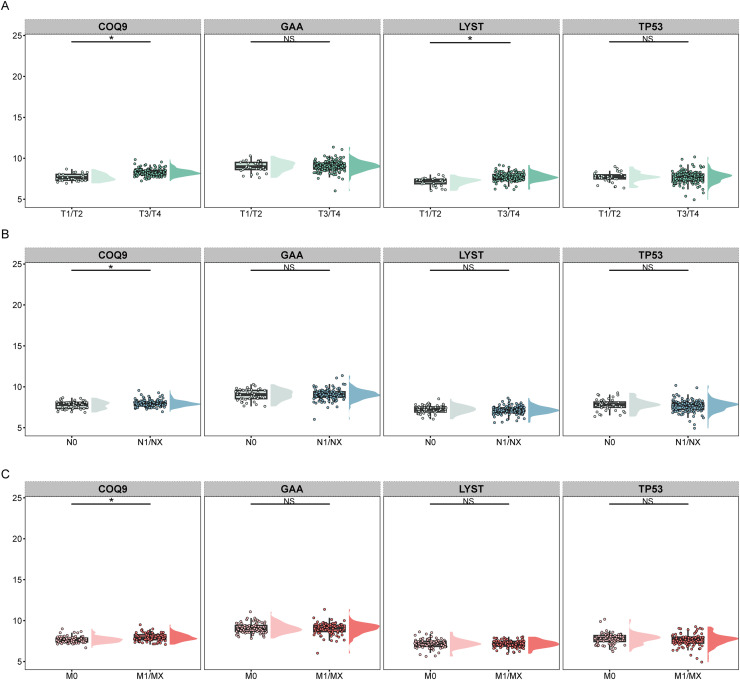
Clinical relevance of core lactylation-related genes in pancreatic cancer. A. Box plots indicating the association between higher expression of COQ9 and LYST and advanced T stages in pancreatic cancer. B. Box plots showing the correlation between higher expression of COQ9 and advanced N stages in pancreatic cancer patients. C. Box plots revealing that higher COQ9 expression is associated with more advanced M stages in pancreatic cancer.

In terms of clinical information, higher expression of COQ9 and LYST were associated with later T stages (Fig. 5A). Higher expression of COQ9 was associated with later N stages (Fig. 5B). Higher COQ9 expression were associated with later M stages (Fig. 5C). Overall, these core lactylation genes are significantly related to the clinical staging of pancreatic cancer patients. Spearman correlation analysis was used to evaluate these associations. Results showed significant positive correlations between T stage and COQ9 (r=0.32, P<0.01) and LYST (r=0.28, P<0.01). COQ9 expression was also positively correlated with N stage (r=0.25, *P* < 0.05) and M stage (r=0.23, *P* < 0.05).

### Immune effects of core genes

**Fig. 6:**
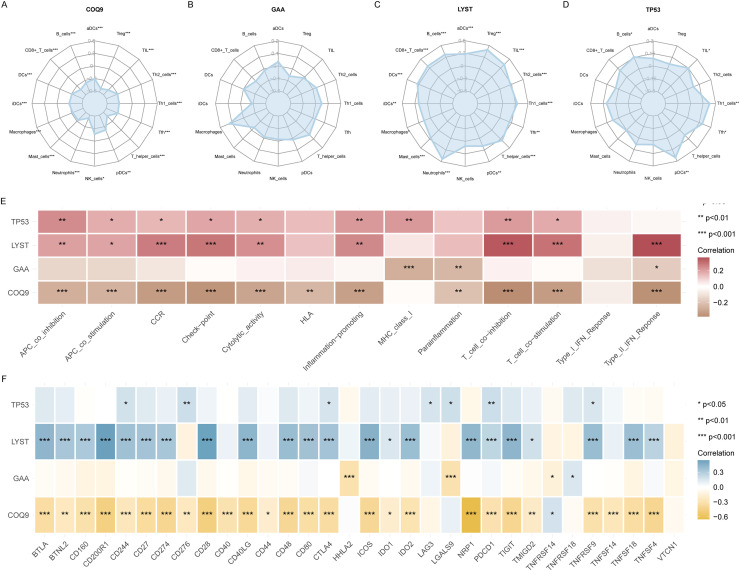
Immune implications of core lactylation-related genes. A-D. Radar charts showing the correlation between core lactylation-related genes (COQ9, GAA, LYST and TP53) and the relative abundance of 16 different immune cell types as assessed by ssGSEA. Each chart highlights the individual gene's significant positive or negative correlation with specific immune cells: A: COQ9 is significantly negative correlated with most immune cells. B: GAA exhibits no statistically significant correlation with most immune cells. C: LYST is significantly positively correlated with the majority of immune cells. D: TP53 shows a significant negative correlation with B cells, TIL cells, Th1 cells, Tfh cells and pDCs cells. E. A heatmap of correlation showing the relationship between core lactylation-related genes and various immune processes, suggesting that TP53 and LYST may act as immune activity genes due to their positive correlation with most immune processes. F. Correlation analysis between core lactylation-related genes and immune checkpoint molecules, indicating that LYST is mostly positively correlated with immune checkpoint molecules, while COQ9 is significantly negatively correlated.

The immune impact of the core genes was evaluated using ssGSEA, which assessed the relative abundance of 16 types of immune cells and calculated the correlation between core genes and these immune cells. The gene sets for the 16 immune cell types were obtained from the 'Immune Cell Signature' collection in the MSigDB database (version 7.5.1). The results showed that COQ9 was significantly negative correlated with most immune cells (Fig. 6A). There was no statistically significant correlation between GAA and most immune cells (Fig. 6B). LYST was significantly positively correlated with the majority of immune cells (Fig.s 6C), while TP53 showed a significant negative correlation with B cells, TIL cells, Th1 cells, Tfh cells and pDCs cells (Fig. 6D). A heatmap of correlations illustrated the relationships between core lactylation genes and immune processes, with LYST and TP53 being positively correlated with most immune processes, suggesting that these two genes might be immune activity genes, while COQ9 was negative correlated with most immune processes (Fig. 6E). Additionally, the expression correlation between core lactylation genes and immune checkpoint molecules was analyzed, LYST was mostly positively correlated with immune checkpoint molecules, while COQ9 was significantly negatively correlated (Fig. 6F).

### Signaling pathways involving core lactylation genes

**Fig. 7:**
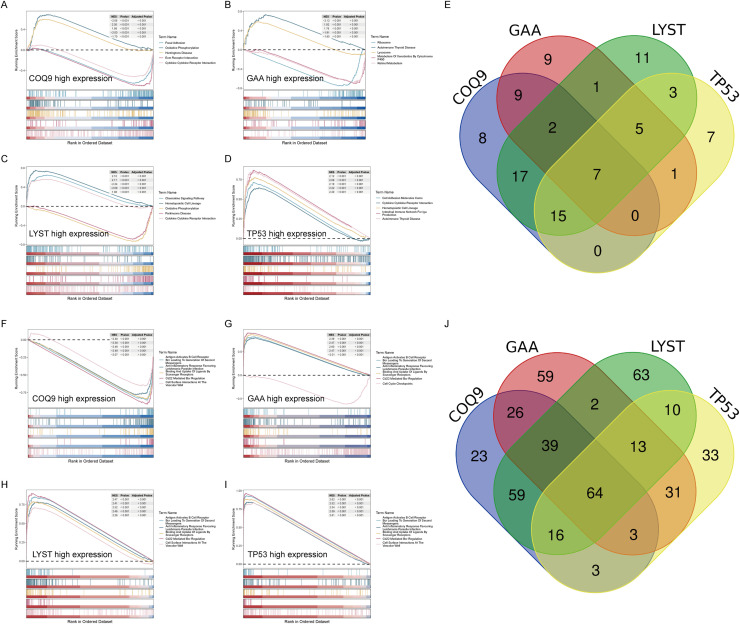
Signaling pathways involving core lactylation-related genes. A-E. Graphs representing the GSEA analysis of the high and low expression groups of the core lactylation-related genes, based on median gene expression. F-J. Analysis of REACTOME signaling pathways showing 64 conserved upregulated pathways across the high expression groups of the core lactylation-related genes, highlighting the breadth of immune and metabolic processes influenced by lactylation. Two PDAC cell lines (MIA PaCa-2 and AsPC-1) were treated with lactate (20 mM) or the glycolysis inhibitor 2-DG (10 mM). A-B. Lactylation levels in PDAC tissues and adjacent para-carcinoma tissues were visualized by IHC staining in tissue microarrays. scale bar = 200 μm. C-D. Transwell assay results showed that lactate treatment enhanced the invasive ability of PDAC cells, whereas 2-DG treatment significantly reduced cell invasion. scale bar = 100 μm. E-F. Colony formation assays demonstrated that lactate promoted PDAC cell proliferation, while 2-DG inhibited colony formation ability. G-H. Scratch wound healing assay results: representative images of all groups at 0 and 24 h post-wounding. Lactate accelerated wound healing (enhanced migration), while 2-DG delayed this process (impaired migration). scale bar = 100 μm. All data are presented as mean ± SD. Statistical analysis was performed by ANOVA followed by Dunnett’s multiple comparisons test. *P < 0.05, **P < 0.01, ***P < 0.001.

The signaling pathways involving core lactylation genes were analyzed by dividing samples into high and low expression groups based on the median gene expression, followed by GSEA enrichment analysis of KEGG pathways. The results showed that the high expression groups of the 4 genes shared 7 common pathways: KEGG_ASTHMA, KEGG_INTESTINAL_IMMUNE_NETWORK_FOR_IGA_PRODUCTION, KEGG_AUTOIMMUNE_THYROID_DISEASE, KEGG_LEISHMANIA_INFECTION, KEGG_CELL_ADHESION_MOLECULES_CAMS, KEGG_PROTEASOME, KEGG_TYPE_I_DIABETES_MELLITUS, indicating that these pathways might be conserved upregulated pathways of core lactylation genes (Fig. 7A-E). An analysis was also conducted on the REACTOME signaling pathways, identifying 64 conserved upregulated pathways (Fig. 7F-J).

### Lactate promotes while glycolysis inhibitor 2-DG suppresses proliferation and migration of PDAC cells

PDAC cells (MIA PaCa-2, AsPC-1) were allowed to attach for 24 h, then treated with 20 mM lactate or 10 mM 2-DG for 48 h before subsequent assays. In pancreatic tissues from patients with PDAC, lactate levels were significantly higher compared to adjacent normal pancreatic tissues (Fig.s S1A-B). To clarify the regulatory role of glycolysis in histone lactylation, we treated PDAC cells with lactate (a glycolysis-derived metabolite) and the glycolysis inhibitor 2-deoxy-D-glucose (2-DG). Our results showed that lactate treatment promoted PDAC cell invasion, colony formation, and migration, whereas 2-DG administration significantly inhibited these malignant biological behaviors (Fig.s S1C-H).

## Discussion

The pivotal role of metabolic reprogramming in cancer's progression has garnered extensive attention, with lactylation emerging as a novel post-translational modification with significant implications in oncology [21]. Our study's findings underscore the complexity of lactylation's role in pancreatic cancer, presenting both congruencies and divergences from existing literature. Notably, our identification of core lactylation-modified proteins and their correlation with disease progression and immune modulation provides novel insights into pancreatic cancer's pathophysiology, aligning with and expanding upon previous research [24]. Core lactylation-modified proteins were defined as those meeting the criteria of log2(fold change) > 2 (upregulated) or < 0.5 (downregulated) with an adj.*P* < 0.01 in both comparison groups.

Comparative analysis with existing studies reveals a nuanced landscape of lactylation's role in cancer. For instance, the upregulation of specific lactylation-modified proteins in our study parallels findings in other cancers, suggesting a conserved mechanism wherein lactylation facilitates cancer cell adaptation to metabolic stress [2]. This is consistent with the work of Zhang et al., who first identified lactylation as a key regulator of gene expression in response to metabolic changes in cancer cells [25]. However, our findings extend this knowledge by pinpointing specific proteins and pathways implicated in pancreatic cancer, thereby offering a more detailed understanding of lactylation's role in this particular malignancy [26].

The correlation between lactylation-modified proteins and immune cell abundance in the tumor microenvironment observed in our study also finds resonance in the literature. The significant positive correlation between specific lactylation-modified proteins and the presence of immune cells such as TILs and B cells echoes the emerging consensus that metabolic reprogramming, including lactylation, can significantly influence the immune landscape of tumors [15]. This is in line with recent studies suggesting that lactate, the precursor of lactylation, can modulate the immune microenvironment, potentially impacting immunotherapy's efficacy [27]. Our findings further contribute to this discourse by elucidating the specific lactylation-mediated pathways that may modulate immune cell function in pancreatic cancer.

However, our study also presents findings that diverge from the existing literature, particularly regarding the role of certain lactylation-modified proteins in cancer progression. While some studies have highlighted the pro-tumorigenic role of lactylation, our data suggest a more complex picture, with certain lactylation-modified proteins showing potential anti-tumorigenic properties [28]. This discrepancy underscores the context-dependent nature of lactylation's role in cancer, suggesting that the impact of lactylation on tumor biology may vary significantly across different types of cancer and even within different stages of the same cancer [29].

The clinical implications of our findings are profound, particularly concerning the identification of novel biomarkers and therapeutic targets. The significant correlation between specific lactylation-modified proteins and clinical stages of pancreatic cancer underscores the potential of these proteins as biomarkers for disease progression [15]. Furthermore, the positive correlation between certain lactylation-modified proteins and the efficacy of immune checkpoint inhibitors suggests a potential avenue to enhance immunotherapy's effectiveness in pancreatic cancer, a malignancy notoriously resistant to current immunotherapeutic strategies [2].

Looking ahead, our study opens several avenues for future research. The detailed elucidation of the mechanisms by which lactylation-modified proteins influence pancreatic cancer progression and the immune response warrants further investigation [24]. Additionally, the potential therapeutic implications of targeting lactylation pathways in pancreatic cancer, either alone or in combination with existing treatments, present a promising area for exploration [21]. Finally, the role of lactylation in the tumor microenvironment, particularly in the context of immune evasion and response to therapy, remains an intriguing subject for further study [24].

In conclusion, our research contributes to the growing body of literature on lactylation in cancer, providing novel insights into its role in pancreatic cancer specifically. By juxtaposing our findings with existing studies, we highlight both the corroborative and unique aspects of our research, underscoring the complex interplay between lactylation, cancer progression, and the immune response. As we advance our understanding of this intricate landscape, the translational potential of targeting lactylation in pancreatic cancer holds promise for developing more effective diagnostic and therapeutic strategies, offering new hope for patients battling this devastating disease.

## Ethics approval statement

This study was approved by the Ethics Committee of Hangzhou First People's Hospital (Approval No. ZN-2024293-02). All procedures involving human participants were conducted in accordance with the Declaration of Helsinki, and informed consent was obtained from all participants or their legal representatives. Human pancreatic tissue samples were preserved in RNA Later solution (Thermo Fisher) and stored at -80°C to prevent degradation.

## CRediT authorship contribution statement

**Dong Xu:** Writing – original draft, Writing – review & editing, Funding acquisition, Conceptualization. **Qiuyang Chen:** Writing – original draft, Formal analysis, Data curation, Conceptualization. **Liang Wang:** Resources, Investigation, Formal analysis, Data curation. **Liangliang Li:** Resources, Investigation, Data curation. **Kangwen Cheng:** Resources, Investigation, Funding acquisition, Data curation, Conceptualization. **Xinchun Liu:** Conceptualization, Supervision, Writing – review & editing. **Kai Lin:** Writing – original draft, Writing – review & editing, Funding acquisition, Formal analysis, Data curation.

## Declaration of competing interest

All authors declare that they have no known competing financial interests or personal relationships that could have appeared to influence the work reported in this paper.

## Appendix Supplementary materials

Image, application 1
