# Supplementary figures and images for: Lactylation profiling reveals novel biomarkers and immune interactions in pancreatic cancer
Source: Transl Oncol. 2026 Feb 23;66:102710. doi: 10.1016/j.tranon.2026.102710 (PMC13080600; doi:10.1016/j.tranon.2026.102710)

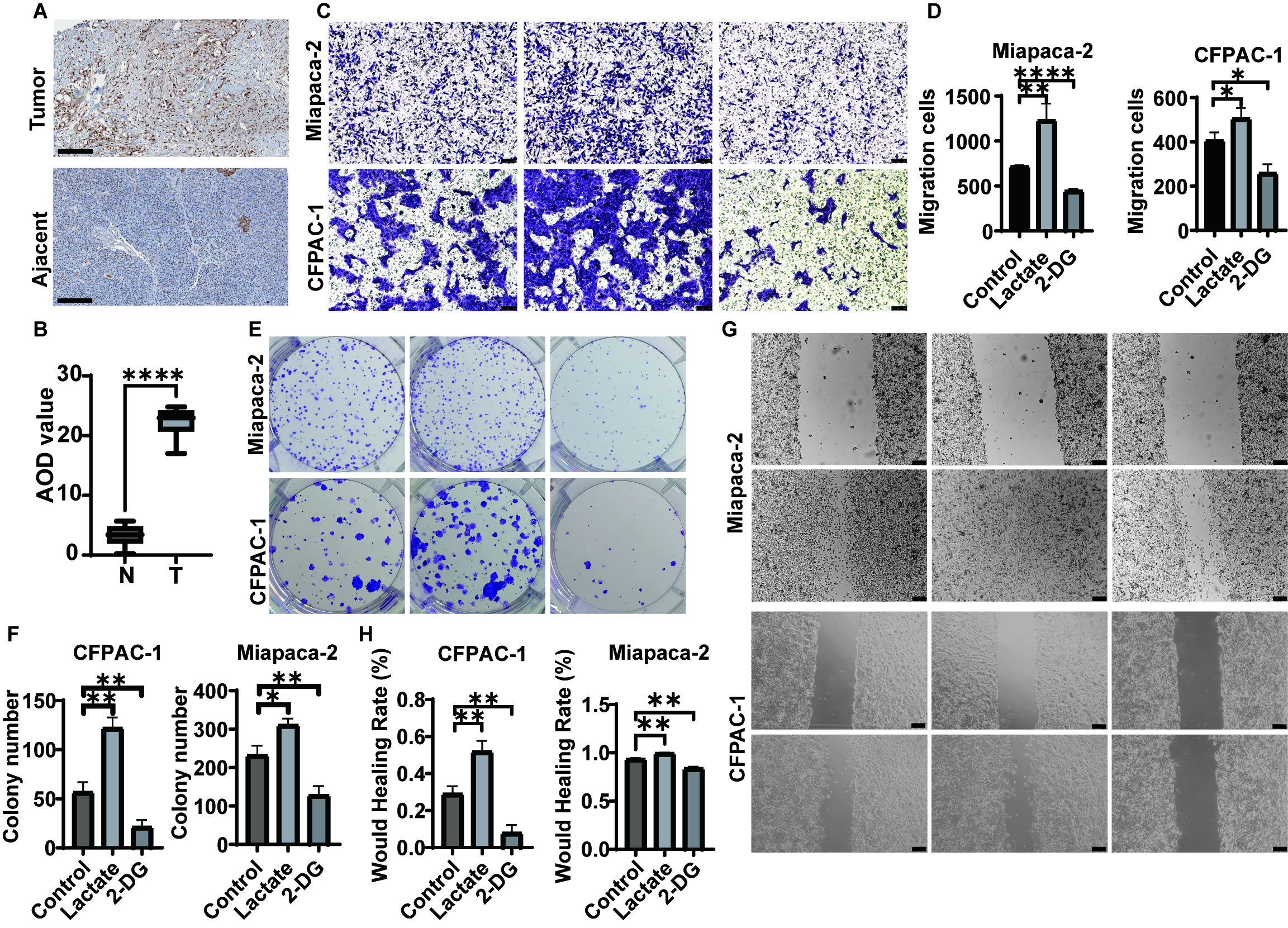

Supplement: Supplementary file 1 [file mmc1.zip › mmc1.tif]
